# Managing Climate Change Refugia for Climate Adaptation

**DOI:** 10.1371/journal.pone.0159909

**Published:** 2016-08-10

**Authors:** Toni Lyn Morelli, Christopher Daly, Solomon Z. Dobrowski, Deanna M. Dulen, Joseph L. Ebersole, Stephen T. Jackson, Jessica D. Lundquist, Constance I. Millar, Sean P. Maher, William B. Monahan, Koren R. Nydick, Kelly T. Redmond, Sarah C. Sawyer, Sarah Stock, Steven R. Beissinger

**Affiliations:** 1 U.S. Geological Survey, DOI Northeast Climate Science Center, Amherst, MA, United States of America; 2 Department of Environmental Science, Policy and Management, University of California, Berkeley, CA, United States of America; 3 Museum of Vertebrate Zoology, University of California, Berkeley, CA, United States of America; 4 College of Engineering, Oregon State University, Corvallis, OR, United States of America; 5 College of Forestry and Conservation, University of Montana, Missoula, MT, United States of America; 6 U.S. National Park Service, Devils Postpile National Monument, Mammoth Lakes, CA, United States of America; 7 U.S. Environmental Protection Agency, Western Ecological Division, Corvallis, OR, United States of America; 8 U.S. Geological Survey, DOI Southwest Climate Science Center, Tucson, AZ, United States of America; 9 Department of Geosciences and School of Natural Resources and Environment, University of Arizona, Tucson, AZ, United States of America; 10 Department of Civil and Environmental Engineering, University of Washington, Seattle, WA, United States of America; 11 USDA Forest Service, Pacific Southwest Research Station, Albany, CA, United States of America; 12 Department of Biology, Missouri State University, Springfield, MO, United States of America; 13 USDA Forest Service, Forest Health Technology Enterprise Team, Fort Collins, CO, United States of America; 14 U.S. National Park Service, Sequoia & Kings Canyon National Parks, Three Rivers, CA, United States of America; 15 Western Regional Climate Center, Desert Research Institute, Reno, NV, United States of America; 16 USDA Forest Service, Pacific Southwest Region, Vallejo, CA, United States of America; 17 U.S. National Park Service, Yosemite National Park, El Portal, CA, United States of America; University of Porto, PORTUGAL

## Abstract

Refugia have long been studied from paleontological and biogeographical perspectives to understand how populations persisted during past periods of unfavorable climate. Recently, researchers have applied the idea to contemporary landscapes to identify climate change refugia, here defined as areas relatively buffered from contemporary climate change over time that enable persistence of valued physical, ecological, and socio-cultural resources. We differentiate historical and contemporary views, and characterize physical and ecological processes that create and maintain climate change refugia. We then delineate how refugia can fit into existing decision support frameworks for climate adaptation and describe seven steps for managing them. Finally, we identify challenges and opportunities for operationalizing the concept of climate change refugia. Managing climate change refugia can be an important option for conservation in the face of ongoing climate change.

## Introduction

Contemporary climate change is occurring in a world pervasively altered by human activities, multiplying challenges for conservation and resource management [1]. To meet these challenges, scientists and natural resource managers are working together to develop guidelines for climate adaptation [2, 3]. A focus on areas resistant to ongoing climate change is increasingly suggested as a potential conservation strategy [4], yet lack of clarity on how to identify and manage these “climate change refugia” hampers practitioners [5].

Here we situate the concept of refugia in the context of contemporary climate change and define steps to manage these areas. Our goals are to: (1) build on recent literature to elaborate the value of climate change refugia as a short- to medium-term management strategy; (2) aid identification of climate change refugia by describing the processes that create them; and (3) introduce a framework for operationalizing the concept of refugia for climate adaptation.

## Evolution of the Refugia Concept as a Climate Adaptation Tool

The concept of climatic refugia has been invoked for nearly two centuries to explain patterns of species distributions in the context of past climatic changes [6]. Understanding how Pleistocene refugia functioned in biodiversity maintenance and as sources for post-glacial re-colonization is important for developing a robust theory for contemporary conservation. We briefly review the theoretical framework for refugia, based in the Quaternary literature but with application to conservation (also see [7, 8, 9]).

19^th^ and 20^th^ century paleoecologists looked to climate refugia to account for poleward and upward species migrations following glacial retreat [6]. Populations persisted and survived during the last glacial maximum in habitats where climate change was buffered or compensated, and in regions where suitable climates were displaced toward the equator or to lower elevations relative to their postglacial distribution. Not only did refugia provide a safe haven during periods of unfavorable climate, but they served as sources for colonization following climate warming [10]. Other research has focused on characterizing refugia as “species pumps” that, due to their isolation and persistence, led to the development of new species and acted as biodiversity hotspots [11].

In the 20^th^ century, the idea of a refugium as a place uniquely buffered from the intense cold and fluctuations of glacial climates expanded to encompass not only past glaciations, but also high temperatures, droughts, and fluctuating sea-levels [9]. With contemporary climate change, species are projected to colonize newly suitable habitats, with populations extirpated at some currently suitable sites [12]. At the same time, “relict” populations are projected to persist in some places owing to unique local habitat conditions, including local dampening of regional climate change effects, which can be seen now in isolated remnants of populations that were distributed widely [13]. These refugia exist because of unique physical characteristics (e.g., climate dynamics, topography) that influence local resource attributes (e.g., species persistence, habitat stability). Physically-based definitions of climate refugia emphasize the mechanisms that enable an area to remain buffered from regional influences and usually do not attempt to link refugia to particular ecological components, such as species or habitats [7]. Biogeographic approaches to refugia emphasize the stability of favorable future habitat [4]. Although this approach is primarily based on correlative species distribution models (SDMs) [14], Dobrowski [15] argued for mechanistic research to expose interactions between refugial terrain, climatic processes, and species distributions.

Building on these approaches, we consider **climate change refugia** as *areas relatively buffered from contemporary climate change over time that enable persistence of valued physical*, *ecological*, *and socio-cultural resources*. This definition applies paleoecological and biogeographical perspectives of refugia [15, 16] to the context of adaptation to the anthropogenic climate change of the 20th century onward. We also emphasize “valued” resources for managers, who are often bound by both policies and public, place-based resource priorities and because, ultimately, all conservation is value-driven [17]. Climate change refugia are at least large enough to sustain a manageable unit of the focal resource, such as a small population or metapopulation [7], rather than smaller, transient micro-environmental “refuges” from exposure and disturbance [9, 18].

This practical approach to refugia integrates physical and ecological perspectives that are often considered separately, and also socio-cultural elements that have not been addressed previously. Physical resources that might be buffered from the effects of climate change include wetlands and snowfields that feed persistent springs [18]. Examples of buffered cultural resources include seasonally frozen waterbodies for ice fishing and skating [19], areas to conduct traditional agriculture and husbandry [20], and archeological sites that continue to be protected from weathering by ice or sea-level rise [21]. Although our perspective and examples here are primarily ecological, we hope that social and physical scientists will be motivated to explore the idea further.

## What Physical and Biological Processes Create Climate Change Refugia?

**Fig 1:**
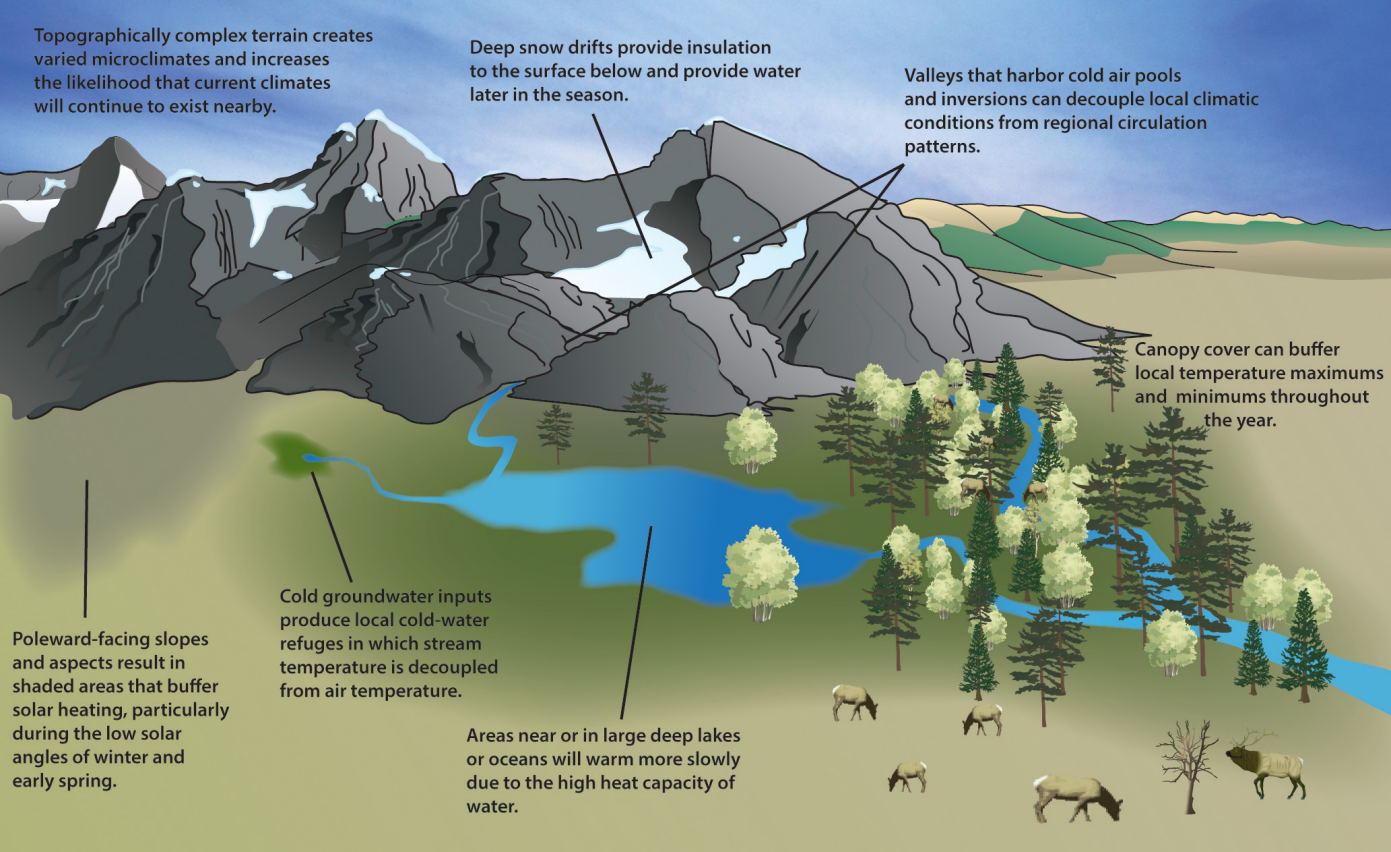
Examples of the physical basis for geographic locations likely to experience reduced rates of climate change.

As identification is key to conserving climate change refugia, we begin with a discussion of the processes that create them. Climate change refugia are characterized by the occurrence of relatively stable local climatic conditions that persist over time, despite change at regional and global scales [22]. This can occur from independent or interacting processes that dampen local climatic variability through time or amplify spatial heterogeneity within a region. Below (and in Fig 1) we review representative examples of these processes (also see [23]). How and whether these processes will be maintained as climate changes in the future remain important research questions [24]; in many environments, current modeling techniques are not able to capture fine-scale processes adequately [25]. We suggest that understanding the processes that lead to climate change refugia will be critical for effectively identifying, mapping, and conserving them to meet management objectives as laid out in the framework introduced later in this paper.

Spatial variability in climate driven by topographic and geomorphic diversity, thermal regimes of specific landforms, and other physical processes increases the likelihood that a wide array of local climates will be maintained in the face of regional and global climate change [15, 26, 27]. These strong microclimatic gradients allow for short distance dispersal and movements to compensate for climate changes and thus can act as climate change refugia. Characteristic examples are cold-air pools (CAPs [28]), where temperature inversions are created by concentrated air in valleys and other topographic depressions that is cooler and moister than surrounding uplands. Although most common under clear night skies, still air, and low temperatures, CAPs can become a semi-permanent feature in topographically sheltered areas. Persistent seasonal features, such as inland penetration of coastal fog and low stratus clouds caused by offshore upwelling, can also produce large climatic response gradients over short distances [29].

**Fig 2:**
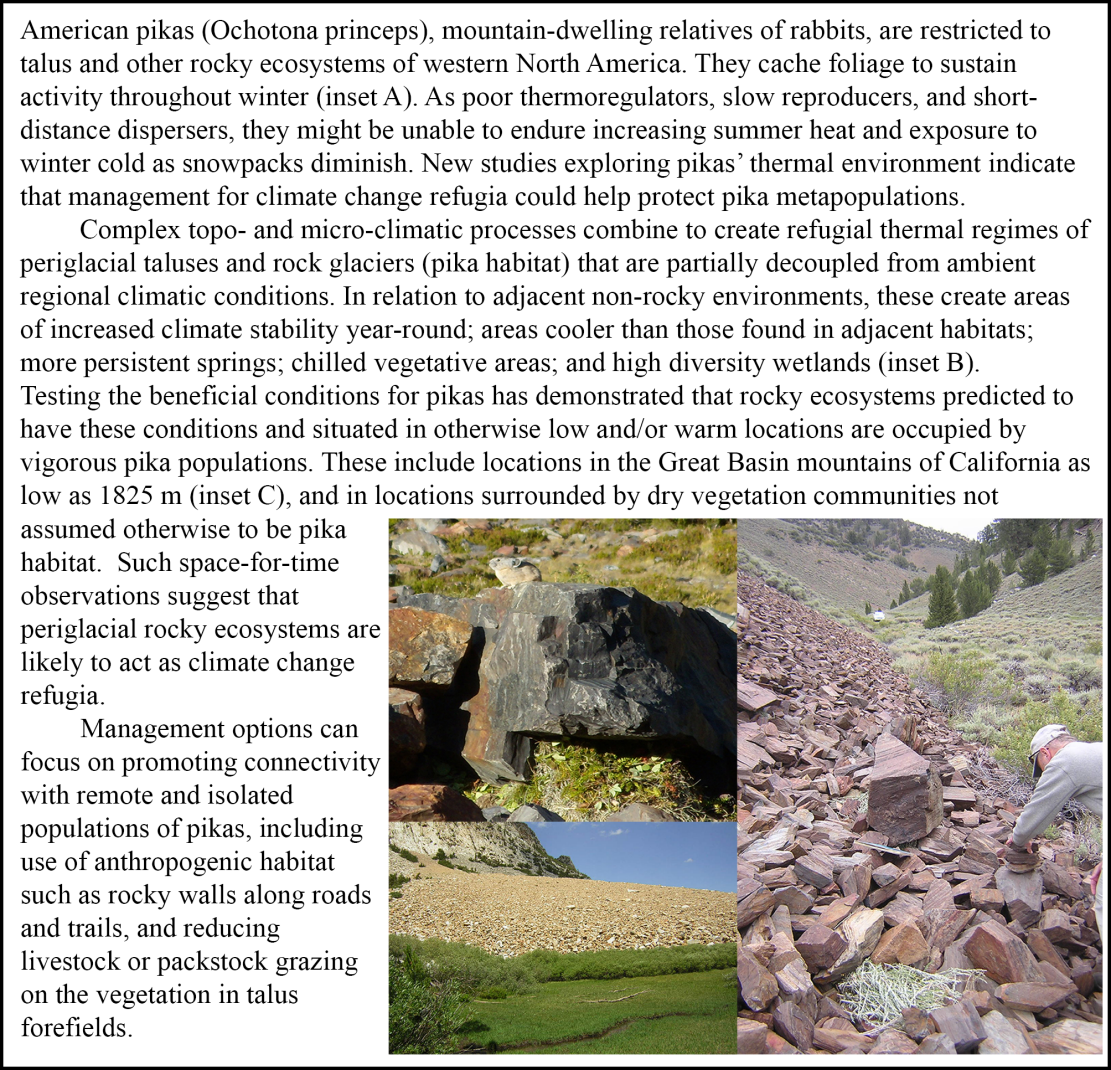
A climate change refugium case study–American pikas and rocky refugial ecosystems. See [35] and references therein. Photos by C. Millar.

Moisture distribution is another common source of spatial heterogeneity as climate changes. Wet areas, including wetlands, riparian zones, rock glaciers, and talus slopes (see American pika case study in Fig 2), can act as climate change refugia [30]. In semi-arid and desert regions, groundwater-fed seeps and springs support persistent populations of highly diverse taxa [31]. Pole-facing slopes generally experience slower hydrologic change [32]. Similarly, deep snow drifts, which can be found in downwind topographic depressions, in granite fissures, or at the base of steep slopes, can serve as important hydrologic reserves. Furthermore, large bodies of water and their surroundings, like coastal areas or deep lakes, are buffered from regional warming because more of the sun’s energy is expended in evaporation than in surface heating [7, 33]. Many of these areas have persisted through the climatic changes of the Holocene, highlighting their capacity to act as distinctive refugial areas. However, loss of moisture or transition from snow- to rain-dominated hydrology might exceed tipping points [34], leading to loss of stability; e.g., glaciers feeding critical water sources that melt out or depletion of groundwater sources from overwithdrawal.

Freshwater climate change refugia for cold-water-adapted species such as salmonids are commonly recognized and increasingly well-studied [33, 36]. Streams and rivers that are buffered from regional air temperatures via cold groundwater inputs from deep aquifers provide cold, sustained streamflows in regions where water temperatures would otherwise become too warm or streamflows too low during the summer months [37]. Such large, cold, connected river networks are recognized as regional strongholds for imperiled fish populations facing increasing pressures from climate warming and other stressors [38]. Other examples of aquatic and marine climate change refugia include areas of cold water within warm river networks [30, 39] and coral reefs thermally-buffered from regional ocean warming by upwelling [40]. Yet, drought and declining base-flows might reduce groundwater inputs into streams, and changes in the geomorphology and riparian vegetation structure of stream reaches can promote or hinder the formation of cold-water refugia [41].

Biologically-mediated processes creating climate change refugia include vegetation effects that can mediate physical disturbance [42] as well as enhance other refugial properties [43]. For example, forest canopies and riparian corridors buffer against climate extremes and variability [44] with consequences for both temperature and water balance. Habitat variability (e.g., variable stand densities, forest gaps, evergreen/deciduous mosaics, riparian corridors) can also increase spatial variability in climate, creating shade and allowing for short-distance dispersal and movements to compensate for climate changes. Areas that are protected from climate-related disturbance, such as increasingly severe fires and extreme floods, also can be considered climate change refugia [45, 46]. Resource managers could consider manipulating these vegetation properties, including structure and fuel loading that lower the risk of increasingly severe fires, to enhance refugial characteristics.

Ecosystem engineers such as beaver and termites that alter water movement and storage and influence the structure and function of heat exchange processes [47] could also contribute to processes creating and maintaining climate change refugia for other species. For example, deep persistent pools created by beavers buffer aquatic species like trout from extreme drought and effects of wildfire [48]. However, biological determinants of climate change refugia are more dynamic than physical processes and might be more transient under climate change.

Other factors will contribute to the capacity of the climate change refugium to act as an effective facilitator of resource persistence [4]. The size of the refugium is important; for example, it should be large enough to support at least some aspect of the resource (e.g., vulnerable life history stage of a population). Furthermore, where the environmental characteristics of the refugium fall on the spectrum of tolerance for the resource is critical; if the refugium is located near a climatic threshold for the resource, it may be a more important focus for management.

## How Can Climate Change Refugia Be Managed?

**Fig 3:**
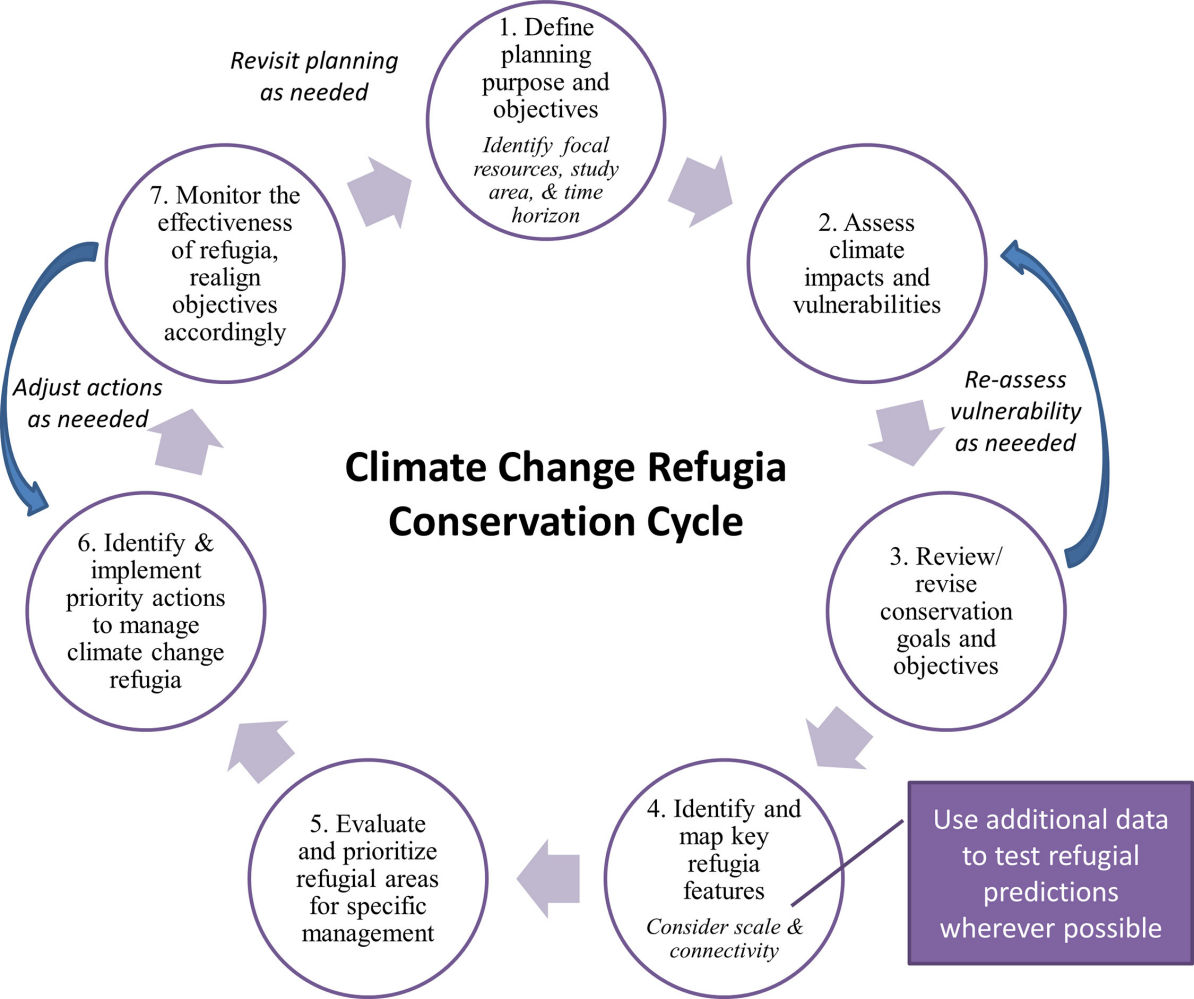
Climate change refugia conservation cycle. See Table 1 for examples.

The idea of regions buffered against climate change has obvious appeal in conservation (e.g., [49]). Managing climate change refugia for local persistence of valued resources gains time for systems to adapt and for managers and society to develop longer-term solutions [2]. We delineate seven steps for managing refugia (Fig 3, Table 1). Our general framework for how to approach climate change adaptation, the climate change refugia conservation cycle (inspired by the climate smart conservation cycle [3]), is spatially explicit and tied to particular actions for managing specific resources. While the examples in Table 1 are ecological, the steps are applicable to physical and socio-cultural resources as well.

**Table 1:** Climate Change Refugia Conservation Cycle with Examples for Identifying, Managing, and Monitoring Climate Change Refugia.

For Habitat (montane meadows) [50]	For Species (bull trout, *Salvelinus confluentus*) [39]
*Step 1 –Define planning purpose & objectives*	
Maintain montane meadow habitat in the Sierra Nevada, with a 15–20 year planning cycle; consider 50–100 year climate projections.	Maintain viable bull trout metapopulations in fragmented Pacific Northwest river networks for 20 years; consider 30–70 year climate projections.
*Step 2 –Assess climate impacts & vulnerabilities*	
Reduced moisture availability and precipitation; disruption of species synchronicity; vegetation shifts; increased recreation impacts from more visitors and longer snow-free seasons.	Changes in hydrologic and thermal regimes, particularly increases in temperature during rearing, spawning, and incubation periods; flow-temperature stresses exacerbated by fire.
*Step 3 –Review/revise conservation goals & objectives*	
Maintain sufficient montane meadow habitats to protect critical ecosystem services in prioritized watersheds.	Maintain viable metapopulations in a subset of historical watersheds over the next 20 years.
*Step 4 –Identify and map key refugia features*	
Potential refugia features: Minimal projected hydrologic and temperature changes, low interannual moisture variability, stable groundwater source, CAPs. Scale is individual interconnecting meadows. Predictions ground-truthed at a subset of sites based on comparisons of historical and current conditions, or based on contemporary monitoring.	Potential refugia features: Groundwater-dependent, connected to supplemental habitats or populations. Scale is hierarchical from basins to critical spawning reaches, with riverine corridors for adults. Test by quantifying responses to fragmentation and climate change, e.g., recent contractions in distribution.
*Step 5 –Evaluate & prioritize refugial areas for specific management*	
Prioritize: Medium or large highly connected meadows; areas of high biodiversity; meadows inhabited by species of management concern now or in the future; areas of high recreational value (if uses are compatible).	Prioritize: Metapopulations exhibiting under-represented diversity; large well-connected networks; groundwater-dominated hydrology; spatial and temporal overlap with other species of concern.
*Step 6 –Identify & implement priority actions to manage climate change refugia*	
Minimize overgrazing; remove encroaching conifers & invasive species; mitigate road & trail impacts; assist migration of lower elevation species; snow fencing to trap snow in desired locations; manage recreation & development; increase connectivity.	Remove or modify barriers (culverts, dams); decommission roads; protect roadless headwaters; assist colonization; manage angling harvest impacts.
*Step 7 –Monitor the effectiveness of designated refugia*, *realign objectives & actions accordingly*	
Monitor: meadow wetness via remote sensing and field measurements; indicator species; downstream watershed variables (streamflow, sediment load, etc.).	Monitor: water temperatures; streamflow; bull trout habitat characteristics and populations.

### Consider target resource needs and vulnerabilities (Steps 1–3)

Similar to the climate-smart conservation cycle [3], the first step of managing climate change refugia is to determine the purpose and scope (Table 1, step 1) by defining the management or conservation target (“valued resource”). The relevant spatial scale can be global, regional, or local; examples include the broader ecoregion encompassing a park or the spatial extent of the focal resource, such as a species’ range. Importantly, the scale of analysis is distinct from the management scale. For instance, landscape connectivity of refugia is important to their effectiveness [4, 51], even though only a small part of the network might be managed. Nonetheless, managers can set measurable objectives that support broader conservation goals.

Assessing vulnerability of the resource to climate change is the next step (Table 1, step 2). Vulnerability assessment considers the *sensitivity* of a resource and measures its *exposure* to particular aspects of, as well its *adaptive capacity* to adjust to, climate change [1, 52]. Key questions to consider include: What aspects of climate change make the resource most vulnerable? What resource characteristics or life history stages might be particularly sensitive to climate change? Does the resource have flexibility to adjust to these changes? Spatially mapping vulnerability factors will help in assessing the potential for local variability.

Following the vulnerability assessment, management or conservation goals should be reevaluated to ensure they remain attainable [3], including whether refugia management is an effective strategy (Table 1, Step 3). Climate change refugia will be most relevant to resources that are moderately to highly vulnerable to climate change on a regional scale, but for which spatial variability in vulnerability factors suggest local buffering of climate change impacts. In addition, climate change refugia management might be a worthwhile strategy for highly valued targets, such as iconic or endemic species, even if they are less vulnerable than other resources. Within a large landscape conservation strategy, projections of population extirpation or other resource loss from a given park might necessitate changing the park management goal from “keeping it common” to “ensuring it remains present”. The revised goals, along with results from the vulnerability assessment (e.g., particular climate change exposure variables yielding high sensitivity), will drive the methods for identifying climate change refugia and mapping them.

### Identify climate change refugia (Step 4)

Where extensive climate and resource data are lacking, first approximations of refugia can be identified based upon the physical and biotic processes that buffer climate change (Fig 1). For example, Ashcroft and colleagues [22] used climatically stable regions within a topographically diverse landscape to predict regions associated with refugial communities. One can also compare current environmental conditions with climate scenarios to map domains that are likely to act as climate change refugia in the future, although this requires high resolution data or in situ measurements [53]. A related method calculates climate change velocity, which uses the distance required to reach a similar climate in the future [54] to identify areas with exceptionally low magnitudes of future change relative to their spatial gradients. Ground-based weather and climate data collection and modeling provide very promising approaches for generating high-resolution regional climate models [55]. However, comprehensive climate data at scales adequate to capture finer-scale refugia for multiple resources and taxa remain elusive due to issues of data continuity, accessibility, and affordability [56, 57]. Moreover, uncertainties regarding biological responses (e.g., acclimatization [58]) remain.

Other methods recognize climate change refugia based on biological data. Past persistence through climate change might be a clue to locations buffered in the future, either for native species or those that will shift into the area [33]. For example, disjunct populations of cool-temperate plant species (e.g., *Tsuga canadensis*) are scattered across Ohio, Indiana, Illinois, and Kentucky, apparent relicts of northward postglacial migrations in the late-glacial or early Holocene. The populations are concentrated in unique microhabitats, usually north-facing slopes and shaded ravines. Similarly, relict populations at the rear or trailing edge of a species’ range might indicate climate change refugia [59]. Moreover, these sites often house important genetic and trait diversity because they are older and perhaps more adapted to conditions at the environmental margin [13]. A systematic inventory of disjunct plant and animal species, accompanied by local habitat characterization and by determination (from biogeography, paleoecology, genetics, or environmental history) of whether they represent relicts or naturalized non-native species, can provide a foundation for broad generalizations on the nature and properties of climate change refugia [60, 61].

One can also identify areas of high genetic diversity or persistence for climate vulnerable species or other resources, which might indicate places where populations have persisted owing to climatic stability or high topographic variability. Morelli and colleagues [50] used occupancy modeling of Belding's ground squirrels (*Urocitellus beldingi*) to identify “anthropogenic” climate change refugia, areas where populations appear to have persisted through time in habitats due to artificial buffering from climate change by food or water supplementation (Table 1). Another method to identify areas that are more resistant to future climatic change is to map the response of a resource to events like warm droughts that are proxies for conditions that will be more common in the future.

**Fig 4:**
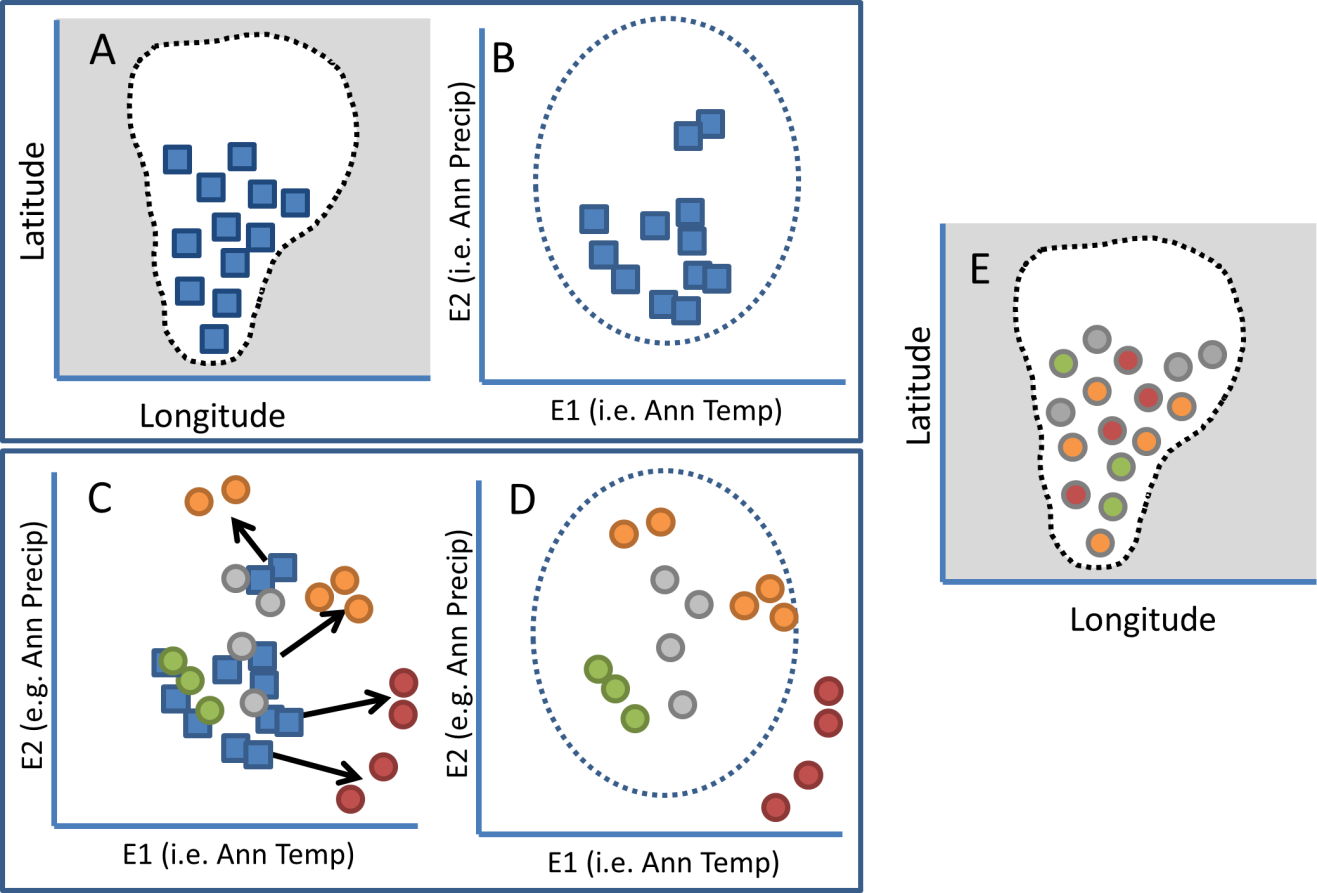
Alternative outcomes of identifying potential climate change refugia from observed and projected climate data using physical or ecological definitions. Observed climate data (e.g., Annual Temperature and Annual Precipitation) from occupied sites (blue squares mapped in geographic space; A) are extracted and plotted (blue squares; B and C). From these same sites, projected climate values are extracted and plotted in climatic space on the same axes (circles; C) to determine the amount of change expected; for a physical perspective, only sites that are expected to change less than a given threshold (green circles) are designated as climate change refugia. For an ecological perspective, information regarding the species environmental limits is modeled or experimentally determined (dotted line; B). Sites that are within this envelope after projected change are considered climate change refugia (green and orange circles; D). Thus, from both the physical and ecological perspectives, green circles are identified as climate change refugia and red circles are not, whereas orange circles are not identified as climate change refugia by the physical definition because they exceed the threshold of change but are designated as such by the ecological definition. Additionally, although currently unoccupied, gray circles represent sites that might be identified as climate change refugia as they are expected to represent suitable environmental conditions under both definitions. The spatial arrangement of climate change refugia and non-refugia (depending upon definition) are mapped in E.

Ecological niche models (ENMs) incorporate occurrences with environmental data to yield an estimate of potential geographic distribution [62]. These correlative approaches essentially combine aspects of the ecological and physical definitions of refugia. Areas where ENMs predict continued persistence under future climate scenarios might indicate climate change refugia (e.g., Fig 4). There are a variety of applications of these techniques to estimate potential shifts in distribution due to climate change [51, 58, 63, 64]. Although they have clear limitations [63], more precise estimates of future suitability can be provided by models that incorporate finer-resolution climate data [65, 66] or consider buffering processes such as groundwater [67]. Overlaying modeled estimates for multiple species or other valued resources can indicate climate change refugia under various scenarios [68]. Different areas could be identified as climate change refugia depending on whether physical or ecological methods are employed (Fig 4). Multiple lines of evidence from combining different approaches can be used to increase confidence in the identification of climate change refugia [63, 64, 69].

An essential though rarely accomplished step is to use independent data to validate potential climate change refugia by testing predictions for specific taxa or ecological characteristics (highlighted as a substep in Fig 3). Potential climate change refugia identified through ecological characteristics (e.g., species diversity or trailing-edge locations) can be examined for topographic or other physical features that enable persistence. The key is to evaluate, as fully as possible, whether a refugial location really meets the needs of the valued resource.

### Prioritize refugial areas and implement management actions (Steps 5–7)

The next step is to prioritize climate change refugia for management. In addition to connectivity, capacity, and size [4, 8, 23, 51, 70], other criteria will be important for prioritization, including representation of valued resources, potential for protecting multiple resources within refugia now and into the future, existing and expected land use change [71], and practical considerations such as the feasibility of management actions and public perspectives. For example, increasing resilience of refugia through costly fuel reduction might be easier in the wildland-urban interface than in remote locations because it would meet multiple objectives, including protection of human communities. Practically speaking, one can overlay the map of potential climate change refugia with maps of other features, vulnerabilities, management potential, and historical legacies or combine refugia maps to identify where overlaps occur. One can also coordinate opportunities and constraints among land owners.

Once locations are prioritized, management options can be identified (Table 1, step 6). Current suites of management tools and actions will need to be analyzed on a case-by-case basis with the best information of future climate and ecological settings to evaluate long-term benefit. Managing for climate change refugia will employ many of the same approaches used to conserve non-refugial targets; ultimately, the climate change refugia framework is a way to prioritize locations for management action given limited resources. What will differ is the emphasis on resistance strategies[72]; protecting, maintaining, and fostering the features and processes of climate buffering identified in the previous steps could include reducing direct and indirect stressors. For example, removing recreation trails through wet meadows to redirect visitor use improves hydrologic function, increases resilience, and could ultimately protect federally listed wetland species. Although some CAPs are protected from human impacts by virtue of their extreme topography and remoteness, others are in valley bottoms which contain trails and roads that might alter hydrology and erosion and aid introduction of pathogens and non-native species. Active fire and fuel management could be prioritized to protect climate change refugia from, or enhance resilience to, extreme fires that otherwise might damage the ecosystem irreversibly. Similarly, managing groundwater by limiting withdrawals and setting minimum ecological flows is relevant for common conservation practice, but storage, pumping, and other active manipulations may become more important options, despite their associated risks.

Unprotected lands identified as climate change refugia could be the focus of acquisitions or easements. If publicly owned, a new area selected for protection specifically for its resistance to climate change could be designated as a climate change refugium via enabling legislation or by another legal or regulatory instrument, or as a “climate change refugia emphasis area” in management plans. For example, National Park Service staff are identifying actions for managing Devils Postpile National Monument’s primary meadow complex, located in a CAP, as a climate change refugium based on ecological and physical characteristics. Potential actions include removing establishing conifers and climate immigrants that disrupt ecosystem function.

**Fig 5:**
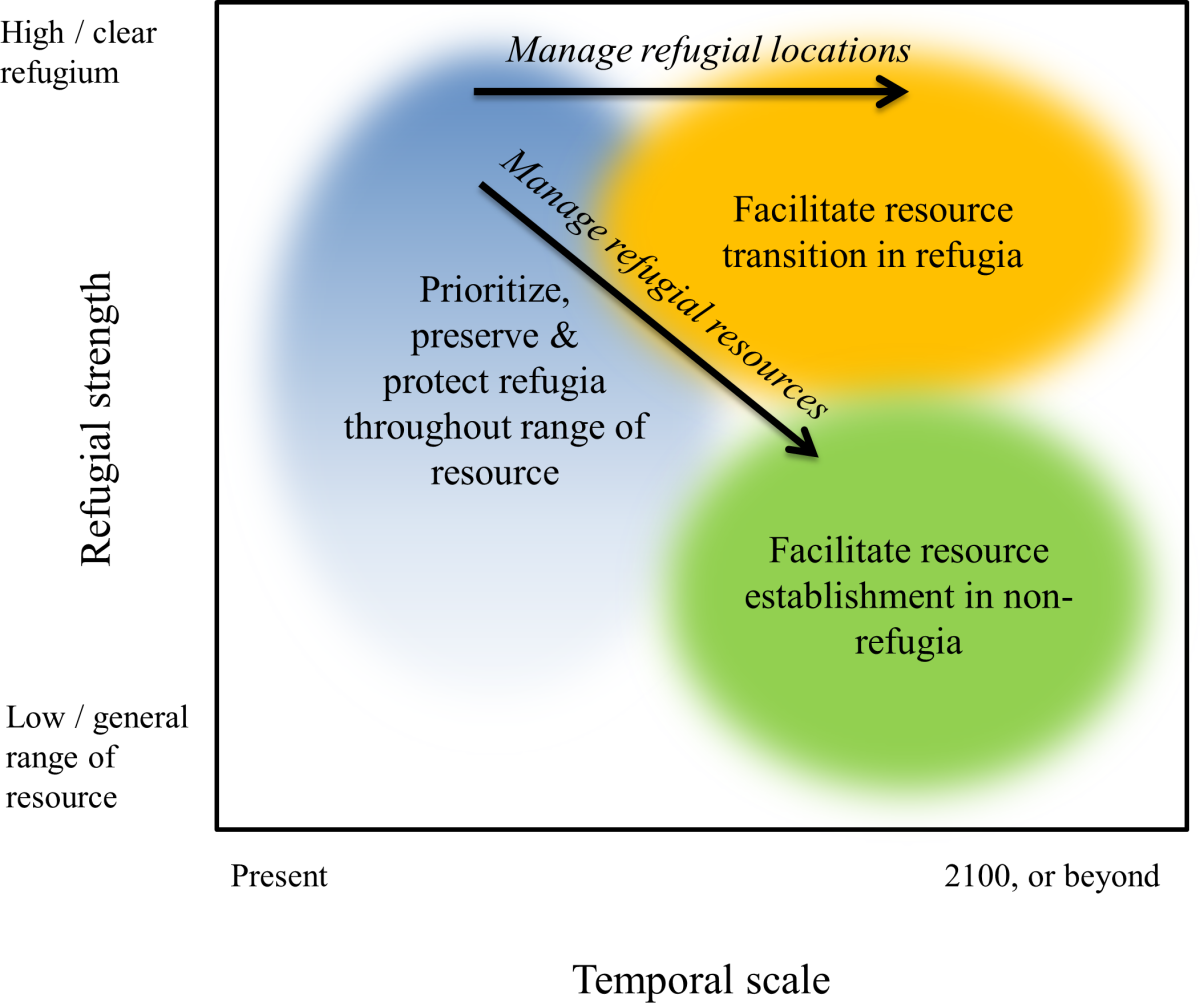
Conceptual model of how climate change refugia might need to be managed differently into the future. Not all areas within the range of the resource (blue) are refugia, and some refugia within the range will persist longer into the future than others (i.e., their refugial characteristics are stronger). Darker blue identifies parts of the range that fall in clearly defined physical refugia, and lighter blue to white are parts of the range that fall in areas with little to no physical refugial value. Prioritization and protection of refugial locations are key management strategies in the near term. Longer term, as climate changes progress beyond the climatic tolerances of the initial refugial resources, refugial locations can be managed for resource transitions (orange) while present-day refugial resources can be promoted elsewhere (green).

One general management action could be to reconnect climate change refugia to each other and nearby non-refugia habitats so as to improve long-term access to refugial areas (Fig 5). This can be done passively by protecting or enhancing connectivity corridors and restoring or protecting nearby non-refugial habitats, or actively by seed banking or captive rearing for future release (i.e., assisted migration). However, reconnection of isolated systems may have adverse outcomes by facilitating the arrival and expansion of competitively superior exotic or even native species and disease vectors. Maintenance of trailing edge refugia could allow persistence of genetic material until longer-term persistence strategies within the shifting range can be established [73]. Existing inter-agency partnerships, such as the U.S. Department of Interior Landscape Conservation Cooperatives, offer opportunities to coordinate and leverage efforts across jurisdictions and despite divergent agency missions.

Given the inherent uncertainty in ways that climate change will affect physical resources, habitats, species interactions, and ecosystem functions, adaptively monitoring the effectiveness of identified refugia and realigning locations and management practices accordingly are critical to the climate change refugia conservation cycle (Table 1, step 7). Millar and colleagues [72] recommended flexible approaches that promote reversible and incremental steps and encouraged ongoing learning and modification. For example, because species vary in their habitat needs, their sensitivity to climate change, and their ability to adapt, climate change refugia will not benefit all species. Decisions might need to be revisited if changes in physical and ecological processes degrade refugium properties, or as management goals change and the protection of other resources becomes more urgent. Depending on the situation, management actions could focus on improving resistance of refugia (e.g., habitat restoration; [30]) or strategies for assisted migration of prioritized species. Monitoring could also ensure that actions taken, such as prescribed burns and increased connectivity, are not increasing the presence of invasive species. Formal decision analysis approaches can aid this process [74].

## What Challenges and Opportunities Exist for Managing Refugia for Climate Adaptation?

As discussed above, there are significant limitations to predicting the duration of climate change refugia. Unlike historical, more cyclical climate contexts, contemporary trends of greenhouse gas emissions indicate long-term directional warming with temperatures predicted to exceed Quaternary levels [75]. Climate change refugia might only be relevant for a certain degree of climatic change, after which conditions in refugia might become climatically stressful for the populations they were designed to protect [29]. Thus, climate change refugia are not necessarily long-term solutions [76]. They function best when coupled with contingency plans, such as tracking geographic shifts in refugial habitats to keep pace with climate change or maintaining genetic material in seed banks, captive propagation, or zoos for future re-introduction.

Another distinction between contemporary climate change and pre-historical processes is the human footprint on air, land, and water [27]. How these factors limit the capacity of climate change refugia will vary, underlining the suggested need to consider dispersal dynamics and intraspecific interactions and to increase habitat connectivity [75]. Challenges also include questions about the scale at which climate change refugia should be identified and managed, uncertainty about the duration of their effectiveness, and confusion over how to incorporate multiple species or other resources that will respond to climate change in different ways. We suggest that an effective climate adaptation strategy must encompass targets that are spatially diverse, temporally dynamic, and multi-faceted. Climate change refugia can be managed from a network approach, considering temporary refugia for incumbents as well as resource transitions and even future refugia for species and other resources previously located elsewhere (Fig 5). Note that the result could be novel community assemblages created by loss of certain species or gain of others that might lead to ecological replacement, as has happened in the past [77]. Again, interagency partnerships, like those facilitated by LCCs, are important for coordinating management and decision-making so that efforts of individual management units complement each other in a regional strategy and the emigration and immigration of resources are recognized by all partners.

Conflicts might arise for management as a result of prioritizing certain resources over others. For example, in Sequoia and Kings Canyon National Parks, endangered frogs are often found in small, climate-sensitive ponds and creeks; non-native trout inhabit former frog habitat in deep high-elevation lakes that could act as climate change refugia [78]. The removal of non-native fish is balanced against their recreational value. Similarly, we suggest that climate change refugia will not be appropriate for conserving all resources. Species already limited to extreme environments, such as alpine species restricted to mountain summits, might not be candidates for management with refugia. On the other hand, some species with extensive home ranges could benefit from climate change refugia; the wide-ranging wolverine (*Gulo gulo*), for example, requires minimum levels of snowpack for den sites that could potentially be managed [65]. Moreover, if regulations or policy require a focus on short-term, immediate protection of high vulnerability areas, limited budgets and staff time might require a tradeoff between managing climate change refugia and other priorities. Managers may need to weigh short-term benefits of conservation of currently limiting habitat against long-term benefits of managing for habitats that are likely to become limiting in the future. For example, under scenarios of increasing resource scarcity (e.g., summer water), trade-offs between future and present needs, and among values resources, will be unavoidable. Ultimately, a mix of strategies, including distributing management actions across areas with a range of climate vulnerabilities, might be the most effective path. The uncertainty inherent to managing climate change refugia is similar to uncertainty involved with all climate adaptation strategies and all areas of risk management and decision-making; it highlights the need for continued integration of physical, biological, and social sciences.

## Conclusion

We have outlined the opportunities and challenges for effective implementation of the climate change refugia concept. Despite the many challenges, managers could use this approach to prioritize areas for climate adaptation where refugial characteristics for a set of valued resources coincide. The concept of resource persistence in refugia under future climate change resonates with managers because it acknowledges opportunities for managers to conserve resources within their protected area as required by enabling legislation and agency policies. Notably, the physical and ecological diversity of landscapes managed by public agencies suggest that they already contain climate change refugia. Thus, this approach provides a way to prioritize land management actions in the face of limited staff and funding. Moreover, there is a great need for such a strategy, even based only on changes in climate that have already occurred. Over 80% of US national parks are already at the extreme warm end of their historical temperature distributions, indicating that ongoing and future changes could transcend temperatures that they experienced over the last century [79]. Implementing science-based management actions to maintain climate change refugia for overlapping resources of value can be an effective way to allocate limited conservation capacity.
