# Correction: Managing Climate Change Refugia for Climate Adaptation

**DOI:** 10.1371/journal.pone.0169725

**Published:** 2017-01-03

**Authors:** Toni Lyn Morelli, Christopher Daly, Solomon Z. Dobrowski, Deanna M. Dulen, Joseph L. Ebersole, Stephen T. Jackson, Jessica D. Lundquist, Constance I. Millar, Sean P. Maher, William B. Monahan, Koren R. Nydick, Kelly T. Redmond, Sarah C. Sawyer, Sarah Stock, Steven R. Beissinger

There is an error in reference 3. The correct reference is: Stein BA, Glick P, Edelson N, Staudt A, editors. Climate-Smart Conservation: Putting Adaptation Principles into Practice. National Wildlife Federation. Washington, D.C.2014.
